# Supplementary material for: Differences in the Expression Levels of SARS-CoV-2 Spike Protein in Cells Treated with mRNA-Based COVID-19 Vaccines: A Study on Vaccines from the Real World
Source: Vaccines (Basel). 2023 Apr 21;11(4):879. doi: 10.3390/vaccines11040879 (PMC10144021; doi:10.3390/vaccines11040879)
Supplement: Supplementary file 1 [file vaccines-11-00879-s001.zip › vaccines-2329878-supplementary.pdf]

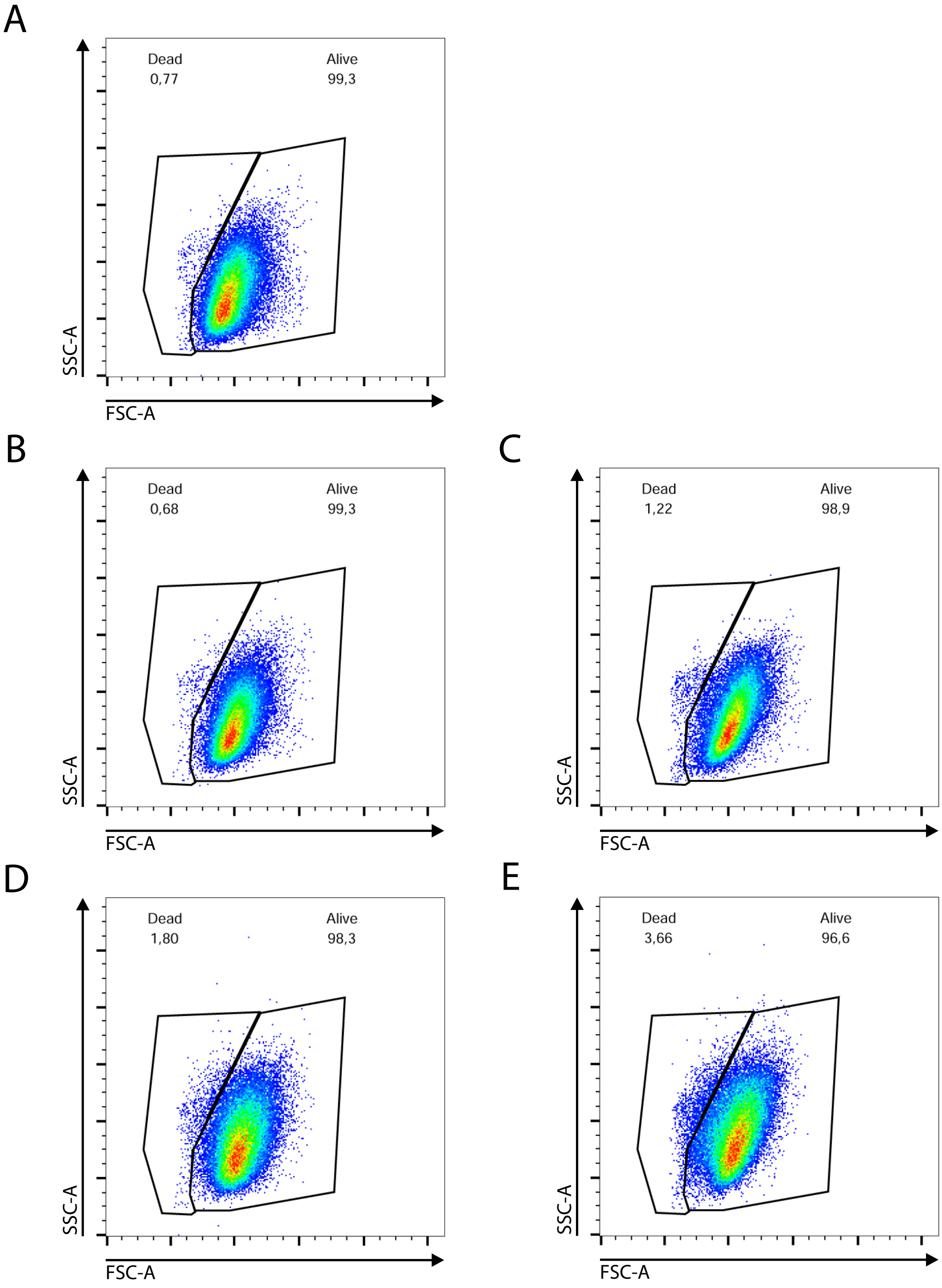

Figure S1. Flow cytometric analysis of Jurkat cell line treated with mRNA-based COVID-19 vaccines

SSC and FSC parameters of untreated (panel A), 1 µl and 10 µl Comirnaty-treated (panels B and C, respectively) and 1 µl and 10 µl Spikevax-treated (panels E and F, respectively) Jurkat cells (representative experiment). The percentage of dead and alive cells is reported.

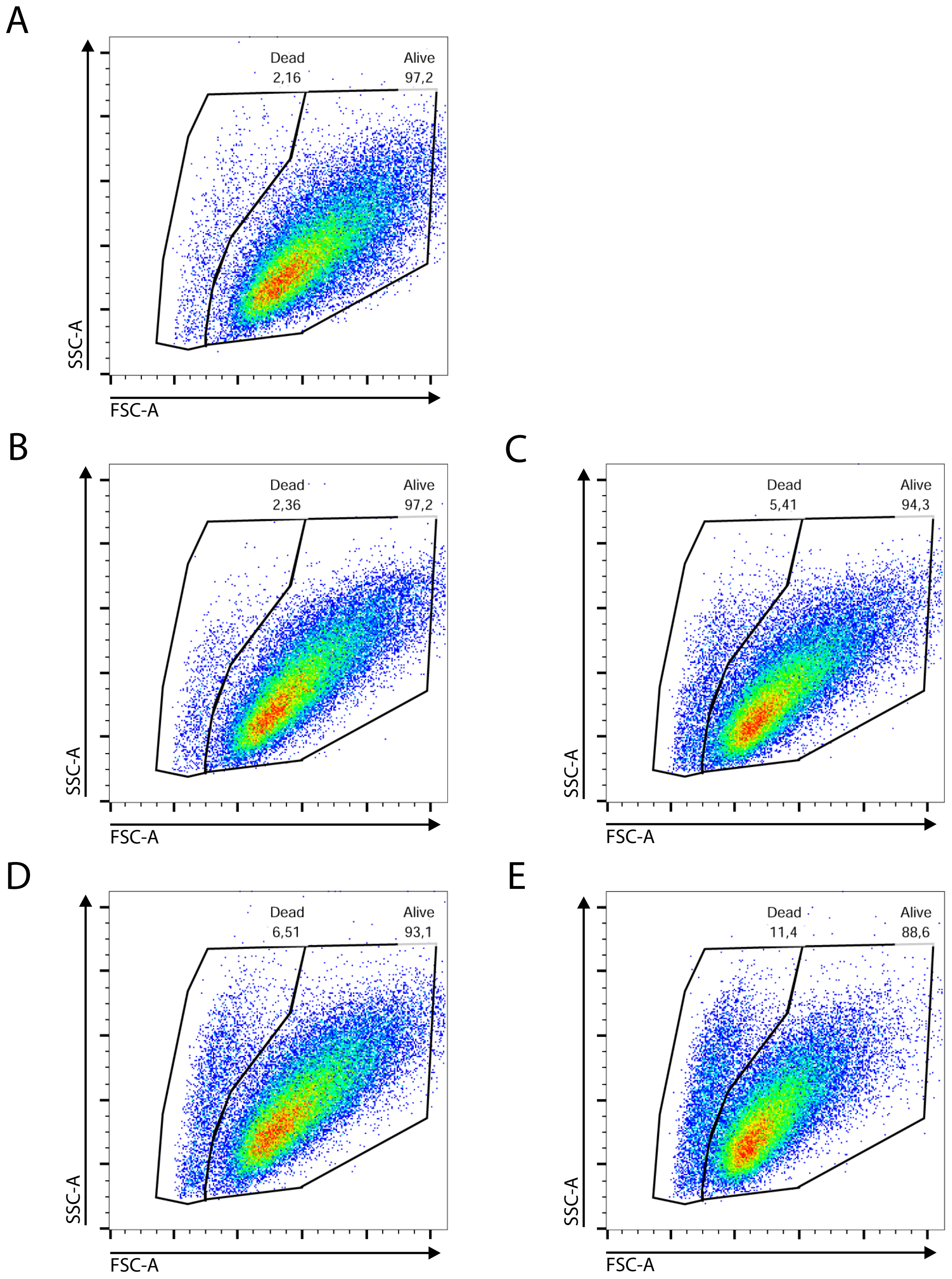

Figure S2. Flow cytometric analysis of K562 cell line treated with mRNA-based COVID-19 vaccines

SSC and FSC parameters of untreated (panel A), 1 µl and 10 µl Comirnaty-treated (panels B and C, respectively) and 1 µl and 10 µl Spikevax-treated (panels E and F, respectively) K562 cells (representative experiment). The percentage of dead and alive cells is reported.
